# Age Related Changes in Metabolite Concentrations in the Normal Spinal Cord

**DOI:** 10.1371/journal.pone.0105774

**Published:** 2014-10-13

**Authors:** Khaled Abdel-Aziz, Bhavana S. Solanky, Marios C. Yiannakas, Daniel R. Altmann, Claudia A. M. Wheeler-Kingshott, Alan J. Thompson, Olga Ciccarelli

**Affiliations:** 1 NMR Research Unit, UCL Institute of Neurology, London, United Kingdom; 2 Department of Brain Repair and Rehabilitation, UCL Institute of Neurology, London, United Kingdom; 3 Department of Neuroinflammation, UCL Institute of Neurology, London, United Kingdom; 4 Medical Statistics Department, London School of Hygiene and Tropical Medicine, London, United Kingdom; 5 National Institute of Health Research (NIHR) University College London Hospitals (UCLH) Biomedical Research Centre (BRC), London, United Kingdom; University of Leipzig, Germany

## Abstract

Magnetic resonance spectroscopy (MRS) studies have previously described metabolite changes associated with aging of the healthy brain and provided insights into normal brain aging that can assist us in differentiating age-related changes from those associated with neurological disease. The present study investigates whether age-related changes in metabolite concentrations occur in the healthy cervical spinal cord. 25 healthy volunteers, aged 23–65 years, underwent conventional imaging and single-voxel MRS of the upper cervical cord using an optimised point resolved spectroscopy sequence on a 3T Achieva system. Metabolite concentrations normalised to unsuppressed water were quantified using LCModel and associations between age and spinal cord metabolite concentrations were examined using multiple regressions. A linear decline in total N-Acetyl-aspartate concentration (0.049 mmol/L lower per additional year of age, p = 0.010) and Glutamate-Glutamine concentration (0.054 mmol/L lower per additional year of age, p = 0.002) was seen within our sample age range, starting in the early twenties. The findings suggest that neuroaxonal loss and/or metabolic neuronal dysfunction, and decline in glutamate-glutamine neurotransmitter pool progress with aging.

## Introduction

Human senescence is associated with deterioration in physical performance which, in part, can be attributed to age-related neurodegeneration of the spinal cord. Decline in motor agility and gait are ubiquitous features of aging which can begin from the fourth decade of life; typically, walking becomes slower, with shortening of stride length and a tendency to stoop [1], [2]. Sensory perception across all the sensory modalities can become impaired [3]–[6], with an increased incidence of bowel, bladder and erectile dysfunction [7]–[10]. Studies of humans and rodents show that advancing age is associated with aberrations of spinal myelin, proliferation of astrocytes and reduced axonal number and diameter within spinal sensory and motor tracts [11]–[13]. Quantitative morphometric studies in humans show that the decrease in dorsal root fibres begins in the third decade of life [14], whilst quantitative MRI has shown that diffusion anisotropy in the upper cervical cord declines with normal aging, with loss of fibre coherence beginning from the age of ten [15].

Conventional MRI of the spinal cord lacks the necessary sensitivity to detect these microstructural changes. T2 hyperintensities which are commonly seen in the aging brain are only rarely seen in the spinal cord [15]–[17] and age-related volume loss appears to be less marked in the spinal cord than in the brain; although some studies have reported a negative correlation between cord cross sectional area (CSA) and age [18], [19], others have found no change in CSA in the elderly [15], [20], [21]. The development of new quantitative MRI techniques, which are more sensitive to change in underlying tissue microstructure and metabolism, may be much more suited to studying aging of the spinal cord *in vivo* [22].

^1^H magnetic resonance spectroscopy (MRS), which allows the quantification of metabolites in human tissue, has been widely used to study healthy aging of the brain in humans [23]–[27] and animals [28]. Over the past decade, developments in imaging acquisition and post-processing, together with the availability of high field scanners, have made it possible to use MRS to study the spinal cord *in-vivo* [29], [30]. Reductions in spinal cord total N-acetylaspartate (tNAA) concentrations are thought to reflect neuroaxonal injury and/or mitochondrial dysfunction in patients with multiple sclerosis, cervical spondylitic myelopathy and amyotrophic lateral sclerosis, [31]–[33] while increases in spinal cord myo-inositol/total creatine (Ins/tCr) ratios in multiple sclerosis and following brachial plexus re-implantation [34], [35] are likely to represent a reactive gliosis. Because metabolite concentrations reflect specific pathological processes, they could potentially become useful imaging biomarkers of the future. Serial MRS investigations in patients with neurodegenerative diseases may therefore be a useful way of monitoring progression and response to treatments. However, periodic imaging is potentially vulnerable to temporal changes in spinal cord metabolites that are associated with normal healthy aging, rather than disease progression and it is, therefore, important to understand how spinal metabolites change with age to improve interpretation of interval changes.

To date no studies of the spinal cord have addressed metabolic changes associated with normal aging. In this study, which was carried out in healthy volunteers, we therefore aimed to (i) investigate whether age was associated with changes in concentrations of commonly quantified metabolites and (ii) explore the effect of gender on metabolite concentrations.

## Materials and Methods

### Study participants

All subjects provided written, informed consent prior to taking part in the research, which was approved by the NRES Committee London Bloomsbury (Formally London REC 2 Ethics Committee).

Healthy volunteers were prospectively recruited from amongst university staff and respondents to adverts within the university and neurology outpatient clinic. A minimum of two subjects per decade of life (between the ages of 20–65) were recruited in order to achieve a good spread of ages. The age of subjects, from youngest to oldest were; 23, 24, 25, 28, 30, 30, 31, 31, 33, 33, 36, 40, 43, 44, 46, 48, 52, 54, 55, 56, 65 and 65 years old. Participants found to have severe spondylitic changes [16], compression of the cord or an intrinsic cord lesion were excluded from the study.

### MRI Protocol

All scans were performed using a 3T Achieva system (Philips Medical Systems, Best, Netherlands), with the manufacturer’s 16-channel neurovascular coil. An MR compatible cervical collar was worn by all volunteers as this has recently been shown to considerably reduce motion artefacts during scanning [36]. All subjects initially underwent conventional structural imaging of the upper cervical cord with: (a) Spin-echo T2-weighted sequence, with TR = 4000 ms; TE = 100 ms; echo train length = 24 echoes; FOV = 160×250 mm^2^; voxel size = 0.6×0.6×3.0 mm^3^;number of excitations (NEX) = 2; 13 contiguous coronal slices, and (b) Dual-echo PD/T2-weighted sequence with TR = 4000 ms; TE = 15/80 ms; FOV = 256×160 mm^2^; voxel size = 1.0×1.0×3.0 mm^3^; NEX = 2; 12 contiguous sagittal slices.

**Figure 1:**
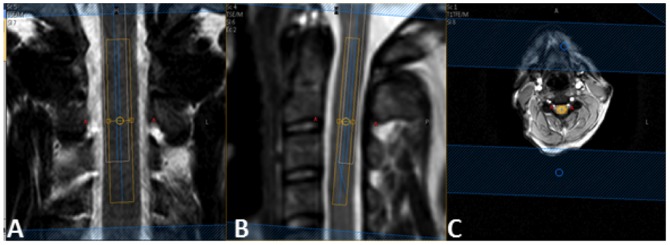
Planning of spectroscopy scans. Coronal (a) and sagittal (b) T2-weighted images of the upper cervical cord in a healthy subject showing voxel placement The NAA voxel (orange) is centred on the C2/3 intervertabral disc, avoiding surrounding CSF. The white voxel illustrates the chemical shift displacement of water. Keeping both the orange voxel (on resonance, 2.02 ppm) and white voxel (4.7 ppm) within the cord, ensures that metabolites between 2.02 and 4.7 ppm (tNAA, tCr, tCho, Glx, Ins) also arise from within the spinal cord and chemical shift displacement of each metabolite need not be an issue. Positioning of the rostrocaudal OVS slabs is shown in periphery of images a+b and positioning of the anterior-posterior OVS slabs is shown in c.

Single voxel MRS data was then acquired using a recently optimised protocol [37]. Cuboid volumes of interest (VOI) with dimensions of approximately 5.4×7.76×55 mm^3^ (2.3 ml) were prescribed and centred on the C2/3 intervertebral disc (Figure 1) using the previously acquired coronal and sagittal T2-weighted and PD/T2-weighted reference scans. The dimensions of the VOI were adjusted in the anterior-posterior (AP) dimension dependent on the size of each subjects spinal cord (mean VOI 2.02 ml; SD±0.22 ml). MRS data was acquired using a point resolved spectroscopy (PRESS) localisation sequence, with triggered iterative shimming, multiply optimized insensitive suppression train (MOIST) water suppression (available on Philips scanners) [38]–[40], 4 outer volume suppression (OVS) slabs (broadband saturation pulses approximately 5 ms duration and 6000 Hz bandwidth, applied twice, sequentially) in the AP and rostrocaudal directions and cardiac gating (TR = 3RR≈3000 ms) using a peripheral pulse unit (350 ms delay from R-wave peak), TE = 30 ms, number of averages = 376. Details of the asymmetric excitation RF pulse and the high-bandwidth refocusing pulse can be found in Table S1. In addition, details of the gradient duration and strength that apply to the default MRS voxel dimension are shown in Table S2.

### Post processing

Metabolite concentrations were quantified using the user-independent LCModel (version 6.3) package [41] and a set of basis spectra simulated using GAMMA [42]. The basis set comprised seventeen metabolites including the macromolecules, specifically, N-acetyl-aspartate (NAA), N-acetylaspartyl glutamate (NAAG), gamma-Aminobutyric acid (GABA), Myo-inositol (Ins), creatine (Cr), phosphocreatine (PCr), choline (Cho), phosphocholine (PCho), glutamate (Glu), glutamine (Gln), glucose, guanidinoacetate, lactate, scyllo-Inositol, taurine, alanine and aspartate. Quantification of metabolites was performed by using the unsuppressed water signal obtained from the same voxel [43]. NAA+NAAG (hereafter, tNAA), choline+phosphocholine (hereafter, tCho), creatine+phosphocreatine (hereafter, tCr), Ins and Glu+Gln (hereafter, Glx) concentrations formed the focus of our analysis. The signal-to-noise ratio (SNR) and full width of half maximum (FWHM) of the tNAA peak provided by LCModel were used to assess spectral quality and Cramér-Rao Lower Bounds (CRLB) values for each metabolite were used to assess the reliability of the spectral fit [37]. Poor quality spectra were excluded from the analysis. Criteria for exclusion were poor water suppression or FWHM>0.13 with SNR<3.

### Statistical analysis

All statistical analyses were performed using IBM SPSS statistical package version 22.0 (IBM Corporation, Armonk, NY, USA). Associations between metabolites and age were examined using linear regression of the metabolite as response variable on age, with gender, linewidth (FWHM) and voxel volume covariates; a quadratic term in age was also entered to examine evidence of non-linearity and removed if p>0.1. Gender differences reported from these models are adjusted for age, linewidth (FWHM) and voxel volume. *P* values of <0.05 were taken to be statistically significant.

## Results

Twenty-five healthy participants were prospectively recruited and scanned. Three participants were excluded from the analysis due to poor spectral quality. Therefore, twenty-two healthy participants (15 females) with a mean age of 40.5 years, standard deviation (SD) 13.1, range 23–65 were included in the final analysis.

**Figure 2:**
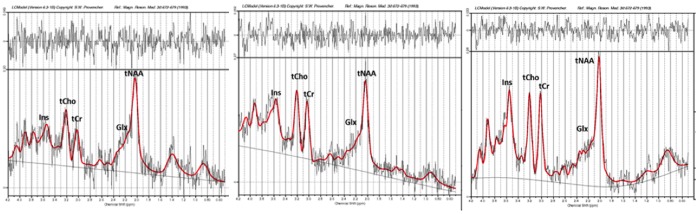
Representative spectra obtained using LCModel from 3 study participants.

Figure 2 shows typical examples of post-processed spectra included in the final analysis. The FWHM and SNR estimated by LCModel (reported as mean ± SD) were 0.11±0.02 ppm and 5.05±1.75 respectively. Cramér-Rao lower bounds (CRLBs) indicated a reliable fit for tNAA, tCho, and tCr. A reliable fit was achieved for Glx in 19 out of 22 spectra and for Ins in 20 out of 22 spectra [44]. Mean CRLBs for each metabolites were; tNAA (7%), tCr (11%), tCho (10%), Ins (11%) and Glx (17%).

**Figure 3:**
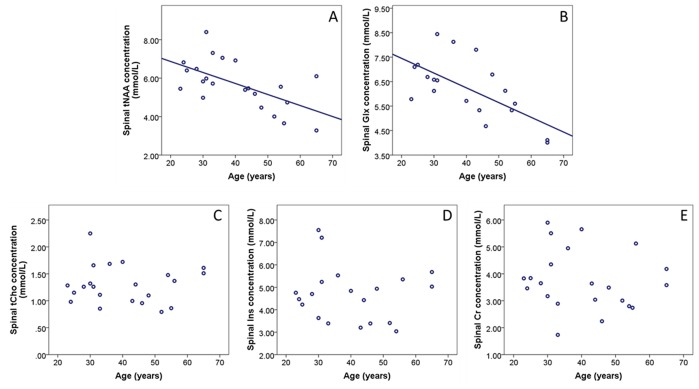
Scatter plots of relationship between age and (A) tNAA, (B) Glx, (C) tCho, (D) Ins and (E) Cr concentrations from the upper cervical cord. Regression lines are shown where there was a significant association (A, B). No significant association was seen between age and tCho, Ins or Cr (C–E).

There was no statistical evidence of a non-linear relationship between metabolite concentrations and age. Older age predicted lower spinal tNAA concentration (0.049 mmol/L lower per additional year of age, p = 0.010) and lower Glx concentration (0.054 mmol/L lower per additional year of age, p = 0.002) (Table 1). Age was not significantly associated with the other metabolites (Figure 3).

**Table 1:** Associations between age (predictor) and metabolite concentrations (response variable).

Association between age and metabolite concentrations				
	Regression coefficient	Standardised regression coefficient	95% CI for regression coefficient	p-value
**tNAA**	−0.049	−0.522	−0.085, −0.013	0.010
**Glx**	−0.054	−0.579	−0.085, −0.022	0.002
**tCho**	0.001	0.026	−0.014, 0.015	0.920
**Ins**	−0.006	−0.067	−0.055, 0.043	0.791
**Cr**	−0.003	−0.040	−0.046, 0.039	0.867

Unstandardised and standardised regression coefficients calculated from the multivariate model are reported with 95% confidence intervals and p-values. The regression models adjusted for gender, linewidth and voxel volume.

Abbreviations: tNAA  = N-acetylaspartate + N-acetylaspartylglutamate, Cr  =  Creatine + phosphocreatine, tCho  =  Choline containing compounds, Ins  =  Myo-inositol, Glx  =  glutamate/glutamine.

Glx concentration was significantly higher in men, mean (SD) 7.27 (0.97) mmol/L, than females, mean (SD) 5.73 (1.05) mmol/L (p = 0.010, adjusting for age, FWHM and voxel volume), but no gender differences were seen with other metabolites (Table 2).

**Table 2:** Mean (SD) water scaled metabolite concentrations derived with LCModel for all subjects and by gender.

Metabolite concentrations (mmol/L) by gender			
	All subjects (*n = 22*)	Male (*n = *7)	Female (*n = 15*)
**tNAA**	5.69 (1.24)	6.05 (1.37)	5.52 (1.18)
**Glx**	6.21 (1.24)	7.27 (0.97)	5.73 (1.05)*
**tCho**	1.30 (0.35)	1.36 (0.53)	1.27 (0.25)
**Ins**	4.70 (1.23)	5.36 (1.83)	4.42 (0.79)
**Cr**	3.76 (1.11)	4.15 (1.47)	3.60 (0.90)

*Significant difference in Glx concentration between male and females (p = 0.010, after adjusting for age, linewidth and voxel volume).

Abbreviations: tNAA  = N-acetylaspartate + N-acetylaspartylglutamate, Cr  =  Creatine + phosphocreatine, tCho  =  Choline containing compounds, Ins  =  Myo-inositol, Glx  =  glutamate/glutamine.

## Discussion

We used a single voxel MRS protocol optimised for improved SNR to permit quantification of Glx from the spinal cord [37]. A higher SNR was achieved by employing a longer voxel and increased signal averaging compared to earlier MRS protocols [29], [30], [45], [46]. Although longer voxel lengths can be associated with worsening of B_0_ convergence [29], our other spectral quality indicators (FWHM and CRLB), after elimination of poor spectra, were comparable to those published by other groups [30], [46].

We aimed to evaluate whether age is associated with changes in metabolite concentrations of the upper cervical cord, as is seen in the brain. Using a recently optimised MRS protocol [37], we quantified metabolite concentrations in the cervical cords of healthy subjects aged between 23 and 65. We found that older age was strongly associated with lower concentrations of tNAA and Glx and that there were significantly lower Glx concentrations in female subjects compared to males.

NAA is a non-essential amino acid which is synthesised by neuronal mitochondria and found exclusively in neurones and their processes [47]–[50]. In the spinal cord, axonal numbers closely correlate with NAA levels quantified by immunoassay [51], and NAA levels decrease in the presence of inhibitors of complexes I, III, IV and V of the mitochondrial respiratory chain [52]. Therefore, in MRS studies, concentrations of tNAA are commonly interpreted as reflecting neuroaxonal integrity and/or mitochondrial energy production [50]. In the current study, we observed a linear decrease in tNAA concentrations in the upper cervical cord with aging, and therefore hypothesise that the tNAA decline reflects age-related neuroaxonal loss and mitochondrial dysfunction. In fact, mitochondrial DNA (MtDNA) deletions and point mutations accumulate during normal CNS aging [53], [54] and, together with increased production of reactive oxygen species [55]–[57], are thought to be responsible for age-related neuroaxonal degeneration. As our sample age range starts at 23 years, we have not been able to ascertain whether age-related changes in the spinal cord occur before the early twenties. Similarly, it is possible that subjects older than 65 could show accelerated decline of tNAA. However, within our sample age range of 23−65, we did not find a quadratic association between age and the concentrations of tNAA and Glx. Future longitudinal studies will study the decline trajectory of tNAA within individuals by following them up for a decade or longer.

Interestingly, in healthy brain aging, reductions in tNAA have been widely reported in grey matter regions [23], [58]–[60], but rarely seen in the white matter [23], [58], [61], [62] which may, in part, be explained by a slower rate of aging-related white matter volume loss in the brain when compared with grey matter [63]. In a previous brain MRS study, a different temporal behaviour of NAA/tCho has been observed between the white matter and grey matter. In the cerebral white matter, the NAA/tCho ratio increases rapidly during the first decade of life before peaking in the second or early third decade, followed by a steady decline starting in the latter half of the third decade of life, whilst in the grey matter, the NAA/tCho ratio enters a steady decline from childhood [64]. Although we have not been able to assess if the age-related decline in tNAA in the spinal cord is also tissue dependent in the current study, due to the difficulty in segmenting white matter and grey matter tissues within the spinal cord, it is possible that tNAA concentration declines faster in spinal grey matter than white matter with age and this could be an area for future research.

Glutamate (Glu), the major excitatory neurotransmitter in mammals plays a major role in the coordination of basic propulsive movement synergy for locomotion at the spinal level [65] and processing and transmitting sensory information in the spinal cord [66]. Glu, as opposed to Glx, which represents a sum of Glu and glutamine, is difficult to measure in the spinal cord. We found that Glx concentration was negatively associated with age. Between 75–86% of the Glx signal is thought to come from Glu [67], and this decline in spinal Glx could largely be explained by neuroaxonal degeneration. As Glu is largely present in neurones at synaptic terminals, with Glu from the extracellular compartment and glial cells considered to be present in very low concentration and therefore contribute very little to the spectroscopy signal [68], [69], it would be expected that Glu (and therefore Glx) will decrease where there is neuronal loss. However, it is interesting that the rate of decline in Glx concentration with age is more rapid than tNAA, which might suggest that the reduction in glutamate-glutamine neurotransmitter pool are driven by more than neuronal loss alone.

The observed association between age and Glx in the spinal cord is in keeping with previous MRS investigations in the brain which have consistently shown declining concentrations of Glu with older age in multiple brain regions including the frontal white matter, parietal grey matter, motor cortex, anterior cingulate cortex, hippocampus, basal ganglia and striatum [24], [26], [68], [70], [71].

An interesting observation in the current study was that of higher concentrations of Glx in men than women. MRS studies in the brain measuring Glx and Glu concentrations have differed on whether gender differences exist. Higher levels have been reported in men compared to women in the parietal grey matter and dorsolateral prefrontal cortex [25], [72], whilst other studies have reported higher concentrations in women in the cerebellum and striatum [73]. Kaiser *et al*. however found no differences in Glu and Gln concentrations between men and women in the corona radiata and mesial motor cortex [68]. Hormonal factors may be responsible for some of the observed gender differences in our study. An examination of the medial prefrontal cortex during the follicular phase and the luteal phase of the menstrual cycle found that Glu/tCr ratios were significantly lower during the luteal phase compared with the follicular phase [74]. Additionally, blood Glu levels vary during the menstrual cycle such that blood Glu levels are inversely correlated to levels of plasma oestrogen and progesterone [75], but interestingly in the work by Zlontik *et al*, Glu levels were significantly higher in men than women at any stage of the menstrual cycle. Although the gender differences in spinal Glx concentrations observed in this study are in keeping with some earlier reports from the brain, further studies, with larger sample sizes will be needed to confirm the validity of this finding and to investigate whether spinal Glx levels vary with female hormone levels.

In the present study no evidence of association was seen between tCho, tCr and Ins concentrations in the spinal cord and age. Although changes have been reported in these metabolites in the brain with aging and have been interpreted as reflecting changes in glial proliferation, those changes are not thought to occur uniformly in the brain, with regional variation commonly reported. tCho concentrations have been reported to be higher in the corpus callosum, parietal lobe, frontal grey matter and pons in older people [23], [27], [62], [76], but remain stable in the frontal, occipital and temporal lobes and the basal ganglia in other studies [60], [77] and decline in the midbrain [23]. Similarly, tCr was seen to increase in the parietal, frontal grey and white matter [27], [62], [78], but other studies found no change in tCr with aging [60], [77]. Much fewer brain studies have assessed Ins levels with aging; a single study found an increase in the frontal grey matter [62], whilst another study reported that Ins/tCr ratios decreased in the frontal grey matter, basal ganglia, and occipital grey matter [79]. A recent meta-analysis of 18 spectroscopy studies assessing regional metabolite changes in healthy brain aging found that there was significant increases in parietal tCho and tCr; although fewer studies assessing changes in Ins had been carried out, levels were not seen to change significantly in the brain with age [27].

To the best of our knowledge, this is the first report of associations between metabolite levels and age within the spinal cord. We have shown a possible effect of aging on tNAA and Glx levels, which should be taken into consideration when planning serial MRS imaging of the spinal cord in clinical and research settings. However, it will take further longitudinal studies to determine the rate of change in metabolites over time in healthy aging and whether metabolite concentrations decline at differing rates in spinal grey matter and white matter. Due to exploratory nature of the study, our sample size was relatively small and absolute metabolite concentrations observed within of our cohort should therefore be interpreted with some caution until future work, using larger sample sizes, further characterises absolute metabolite concentrations by age group. Future experiments should also allow additional scanning time for the inclusion of an experimentally measured macromolecular spectrum, as this has been shown to improve the accuracy of spectral quantification [80]. Studies of brain aging have previously shown that age-related metabolite changes are not uniform and can vary between brain regions, and it is possible that metabolite changes during aging occur at dissimilar rates at different spinal levels which will also require further investigation.

## Supporting Information

Table S1 **Details of RF pulses (default voxel dimensions).** *Maximum B1 of the coil used was 13 µT.
(DOCX)

Table S2 **Details of RF pulses (default voxel dimensions).**
(DOCX)
