# Supplementary material for: Age Related Changes in Metabolite Concentrations in the Normal Spinal Cord
Source: PLoS One. 2014 Oct 13;9(10):e105774. doi: 10.1371/journal.pone.0105774 (PMC4195602; doi:10.1371/journal.pone.0105774)
Supplement: Table S1 — Details of RF pulses (default voxel dimensions). *Maximum B1 of the coil used was 13 µT. (DOCX) [file pone.0105774.s001.docx]

| Table S1. Details of RF pulses (default voxel dimensions) | | | |
| --- | --- | --- | --- |
| Rf Pulses* | Shape | Length(ms) | Bandwidth(Hz) |
| Excitation | ‘spredrex’ | 7.1296ms | 1987Hz |
| Echo | gtst1203 | 6.91ms | 1263Hz |

*Maximum B1 of the coil used was 13 $\mu$T
