# Supplementary material for: Age Related Changes in Metabolite Concentrations in the Normal Spinal Cord
Source: PLoS One. 2014 Oct 13;9(10):e105774. doi: 10.1371/journal.pone.0105774 (PMC4195602; doi:10.1371/journal.pone.0105774)
Supplement: Table S2 — Details of RF pulses (default voxel dimensions). (DOCX) [file pone.0105774.s002.docx]

| Gradients | Strength mT/m | Duration |
| --- | --- | --- |
|  |  |  |
| Spoiler 1 | 5.889 | 8.272 |
| Spoiler 2 | -6.8916 | 7.9396 |
| Spoiler 3 | -0.5782 | 8.222 |
|  |  |  |

Table S2. Details of RF pulses (default voxel dimensions)
